# A detailed macroscopic scoring system for experimental post-traumatic Osteoarthritis in the equine middle carpal joint

**DOI:** 10.1186/s13104-022-06116-x

**Published:** 2022-06-27

**Authors:** Camilla Andersen, Stine Jacobsen, Marie Walters, Casper Lindegaard

**Affiliations:** Department of Veterinary Clinical Sciences, Faculty of Health and Medical Sciences, University of Copenhagen, Højbakkegaard Allé 5, 2630 Taastrup, Denmark

**Keywords:** Cartilage, Osteoarthritis, Gross pathology, Macroscopic pathology, Carpus, Copenhagen Equine Carpal Osteoarthritis Score (CEqCOAS), Copenhagen Equine Total Cartilage Score (CEqTCS

## Abstract

**Objective:**

Osteoarthritis (OA) is a significant health issue in humans as well as horses. Experimental models of equine carpal OA have been used to investigate OA pathogenesis and potential therapeutic candidates. A 5-scale scoring system (OARSI) for macroscopic pathological cartilage changes already exists, but there is a need for a scoring system with better differentiation of severity. The aim of this study was therefore to develop and validate such a scoring system.

**Results:**

New scoring system were developed for cartilage erosions (Copenhagen Equine Total Cartilage Score, CEqTCS) along with synovial membrane pathology and osteochondral fragment healing (Copenhagen Equine Carpal Osteoarthritis Score, CEqCOAS). For the CEqTCS there was excellent intraclass agreement (ICC = 0.993; CI 0.985–0.996; p = 1.08e-31) and consistency (ICC = 0.992; CI 0.985–0.996; p = 4.61e-31), as well as excellent interclass agreement (ICC = 0.974; CI 0.948–0.987, p = 2e-22) and consistency (ICC = 0.973; CI 0.946–0.987; p = 2.77e-22), while the OARSI system had moderate (κ = 0.47) and weak (κ = 0.28) inter- and intra-class agreement, respectively. The OARSI score and the CEqTCS correlated excellently, but every OARSI grade encompassed a wide range of CEqTCS grades. The new score for assessment of equine OA provides means to a better differentiation of OA changes than the existing OARSI system.

## Introduction

Osteoarthritis (OA) is a major cause of pain, disability, and economic costs in humans, leading to a huge effort to both study the disease and investigate potential therapeutic modalities. OA is also a major cause of lameness in horses and economic loss to the equine industry. Since the cartilage of horses and humans have virtually no capacity for intrinsic repair, and because horses and people have comparable cartilage thickness, the horse has been accepted as an excellent model for the study OA and its treatment [1, 2]. One commonly used OA-model is the carpal osteochondral fragment (COF). The COF model entails establishment of an intra-articular chip fracture with an incongruent fragment bed and subsequent continued exercise, and it thus simulates specifically the post traumatic OA phenotype. The COF model results in OA development with moderate lameness, and it has mostly been used for evaluation of OA treatment options [3–10].

To be able to perform accurate and valid evaluation of treatment effects, a sensitive and reliable grading system is required. A method for radiographic scoring of the resulting joint pathology was developed by Smith et al. [11], and guidelines for histopathologic and macroscopic evaluation have been proposed by McIlwraith et al. [12] in corporation with the Osteoarthritis Research Society International (OARSI). The OARSI scoring system for macroscopic pathology is an ordinal 5-scale system, where 0 is normal and 4 is severe changes, and it accounts for the overall gross degeneration of the entire articular surface. However, OA is a complex disease with many facets, where variations in the sort, depth and extent of cartilage damage, changes in the synovial membrane and other specific changes may need to be specifically evaluated. The OARSI system has gained much impact, but in a scoring system with only 5 grades, where each grade embraces both the type, depth and extent of the cartilage damage, detail, and subtle differentiation between various grades of severity is lost.

The aim of this study was to design and validate a new scoring system that would allow grading of macroscopic pathology of OA in the equine COF model in a more detailed manner.

## Main text

### Materials and methods

#### Animals

This study used a convenience sample consisting of detailed 66 photographs of carpal joints from standardbred trotters enrolled in two studies, where OA was induced in one carpus while the contralateral carpus was left as control.

#### OA-induction

The study was carried out using the previously described COF model [3–10]. In brief, an osteochondral fragment was created in the third facet of the radial carpal bone with an 8 mm curved osteotome, and the fragment was left in the joint, partially attached to the parent bone. The horses were stall rested for 14 days after surgery. Starting on day 14 after OA induction, the horses were exercised on a treadmill once a day, 5 days a week as previously described [3–10], leading to development of OA. Horses were euthanized 70 days after surgery.

#### Macroscopic scoring

Macroscopic pathology was scored blindly from detailed photographs (average 12 photographs per joint). The images were scored by two independent observers (CA and STJ). To develop the new score, the two observers scored 5 joints and compared notes to refine the description of each level of the score. This was done to ensure that lesions were described in sufficient detail to obtain the most precise scoring. The joints were both scored according to the OARSI guidelines [12] (Table 1) and according to our new scoring system (Table 1).

**Table 1:** Grading systems to describe macroscopic changes in the equine carpal osteochondral fragment model of post-traumatic osteoarthritis

Score	Region	Range	Parameter	0	1	2	3	4
New CEqCOAS	Cartilage of each carpal bone (RCB, ICB, UCB, 2CB, 3CB, 4CB)	Each: 0–12 Total (= CEqTCS): 0–72	Severity of erosions	None	Partial-thickness erosions, each < 5 mm	Partial-thickness erosions, each > 5 mm	Partial-thickness erosions, each > 5 mm	
			Extent of erosions	None	1–25% of the cartilage surface	26–50% of the cartilage surface	51–75% of the cartilage surface	76–100% of the cartilage surface
	Osteochondral fragment	0–4	Healing and erosions	Full healing with integration with surrounding cartilage (no visible fragment)	Healing non-complete, demarcating border, healthy cartilage around fragment	Fragment attached, mild erosions around fragment	Fragment attached but with severe erosions around fragment	Defect not attached and severe erosions around defect
	Synovial membrane	0–6	Hyperemia	Absent	Mild (only at ac-site)	Moderate	Severe	
			Hyperplasia	Absent	Mild (only at ac-site)	Moderate	Severe	
Existing OARSI cartilage score	Entire middle carpal joint	0–4		﻿No gross fibrillation/ fissuring	﻿Very superficial erosion with articular cartilage swelling	Partial-thickness erosions	Partial and full-thickness erosions	Extensive full-thickness erosions to the level of subchondral bone

In the new macroscopic scoring system, severity and extent of cartilage erosions are scored and multiplied, and a total cartilage pathology score (the CEqTCS, range 0–72) for the joint is calculated as the sum of the products for each carpal bone. Synovial hyperemia and hyperplasia are scored separately and summed (total synovium score, rang 0–6). The osteochondral fragment of the RC is scored and reported separately (range 0–4)

*CEqCOAS* Copenhagen Equine Carpal Osteoarthritis Score, *CEqTCS* Copenhagen Equine Total Cartilage Score, RCB radial carpal bone, *ICB* intermediate carpal bone, *UCB* ulnar carpal bone, *2CB* second carpal bone, *3CB* third carpal bone, *4CB* fourth carpal bone, *OARSI* the Osteoarthritis Research Society International, *ac* arthrocentesis

While the OARSI score encompasses the extent of the cartilage erosions of all articular surfaces in one single score, the new score was developed to assess the articular cartilage of each of the 6 carpal bones individually. The state of the surgically created fragment in the radial carpal bone and the synovial membrane were also scored separately. In each carpal bone, cartilage erosion severity (None = 0; Partial-thickness erosions, each < 5 mm in diameter = 1; Partial-thickness erosions, each > 5 mm in diameter = 2; Full-thickness erosion = 3) and extent of erosions (None = 0; 1–25% of the cartilage surface = 1; 26–50% of the cartilage surface = 2; 51–75% of the cartilage surface = 3; 76–100% of the cartilage surface = 4) were assessed (Table 1). The extent and erosion scores were then multiplied and total cartilage pathology score for the entire joint was calculated as the sum of the products for each of the carpal bones, yielding the Copenhagen Equine Total Cartilage Score (CEqTCS), which ranged from 0–72. The osteochondral fragment of the radial carpal bone was scored separately (Full healing with integration with surrounding cartilage (or no fragment to encompass findings in sham operated joints) = 0; In-complete healing, demarcated border, healthy cartilage around fragment = 1; Fragment attached to parent bone, mild erosions around fragment = 2; Fragment attached to parent bone, but with severe erosions around fragment = 3; Fragment not attached and severe erosions around defect = 4) (Table 1). In the OARSI score, the synovial membrane is evaluated for hypertrophy and inflammation using a 0–4 scale, where 0 represents normal and 4 represent severe pathological changes. In the new score synovial hyperemia and synovial hyperplasia were scored separately (Absent = 0; Mild (only at site of repeated arthrocentesis) = 1; Moderate = 2; Severe = 3). Synovial hyperemia and hyperplasia were totaled for a total synovium score ranging from 0–6. To obtain a total joint pathology score (the Copenhagen Equine Carpal OA Score (CEqCOAS)) the fragment and total synovium scores may be added to the CEqTCS. However, the performance of the CEqTCS, the fragment score and the synovium score were evaluated separately.

Blinded photographs were scored twice by one observer (CA) with a 6-week wash-out period between, and once by the second observer (STJ).

#### Statistical analysis

For quantitative values (CEqTCS) intra- and interclass correlation coefficients for agreement and consistency were calculated using the Finn coefficient two-way model. For categorical/ordinal values (OARSI score, the new fragment score, and the new synovial membrane score) a weighted Cohen’s kappa coefficient was calculated. A Spearman correlation coefficient was calculated between the CEqTCS and the OARSI cartilage score. All analyses were performed using the R statistical software package (Version 1.4.1103, R. RStudio, PBC, Boston, MA).

## Results

A total of 34 carpi, 17 with induced OA and 17 without induced OA were included in the study. Total OARSI scores were between 0 and 4 (median 2.5). Total CEqTCS scores were between 0 and 66 (mean 12.7).

**Fig. 1:**
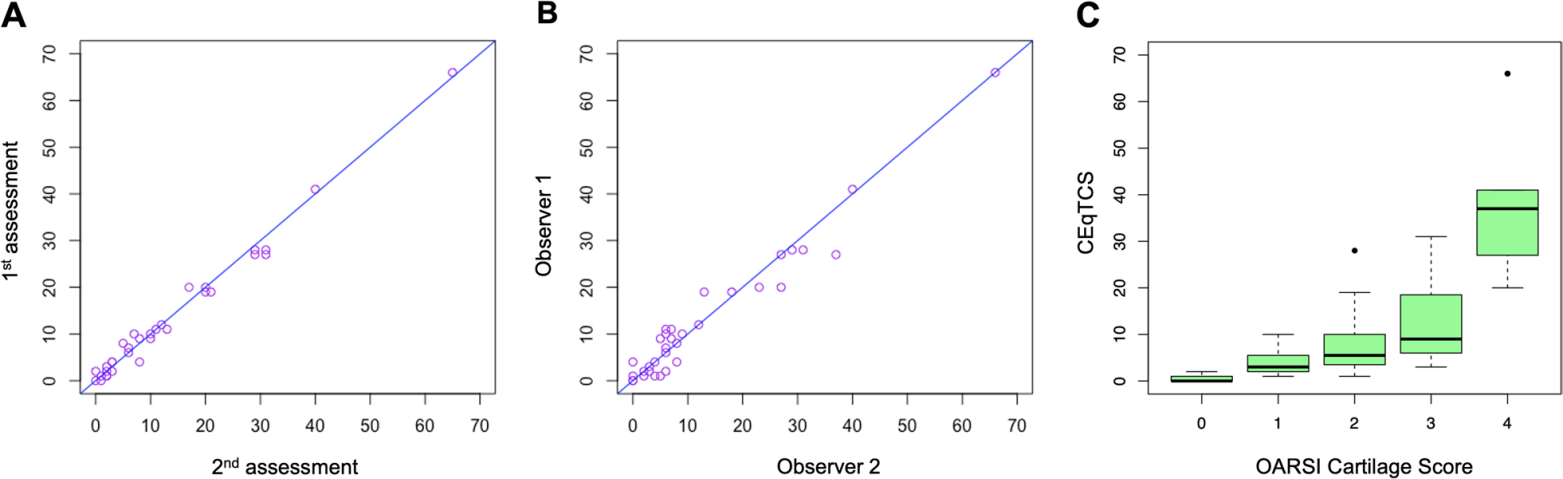
There was excellent intraclass agreement (ICC = 0.993) and consistency (ICC = 0.992) of the new Copenhagen Equine Total Cartilage Score (CEqTCS), when observer 1 scored the pathological changes twice with a 6-week interval **A**. There was excellent interclass agreement (ICC = 0.974) and consistency (ICC = 0.973) (**B**) of the CEqTCS. The CEqTCS correlated excellently with the OARSI score (p = 7.055e-16, r = 0.94) (**C**)

For the OARSI score there was moderate intra-class kappa coefficient (κ = 0.47) and a weak inter-class kappa coefficient (κ = 0.28). For the CEQTCS, there was excellent intraclass agreement (ICC = 0.993; CI 0.985–0.996; p = 1.08e-31) and consistency (ICC = 0.992; CI 0.985–0.996; p = 4.61e-31), as well as excellent interclass agreement (ICC = 0.974; CI 0.948–0.987, p = 2e-22) and consistency (ICC = 0.973; CI 0.946–0.987; p = 2.77e-22) (Fig. 1A, B).

Assessment of fragment healing had excellent intra-class (κ = 0.82) and inter-class (κ = 0.85) kappa coefficient.

Scoring of synovial membrane hyperplasia had moderate intra- (κ = 0.48) and inter-class (κ = 0.50) kappa coefficient; similarly, synovial membrane hyperemia had moderate intra-class (κ = 0.48) and inter-class (κ = 0.50) kappa coefficient. The total synovium score also had moderate intra-class (κ = 0.47) and inter-class (κ = 0.44) kappa coefficient.

**Fig. 2:**
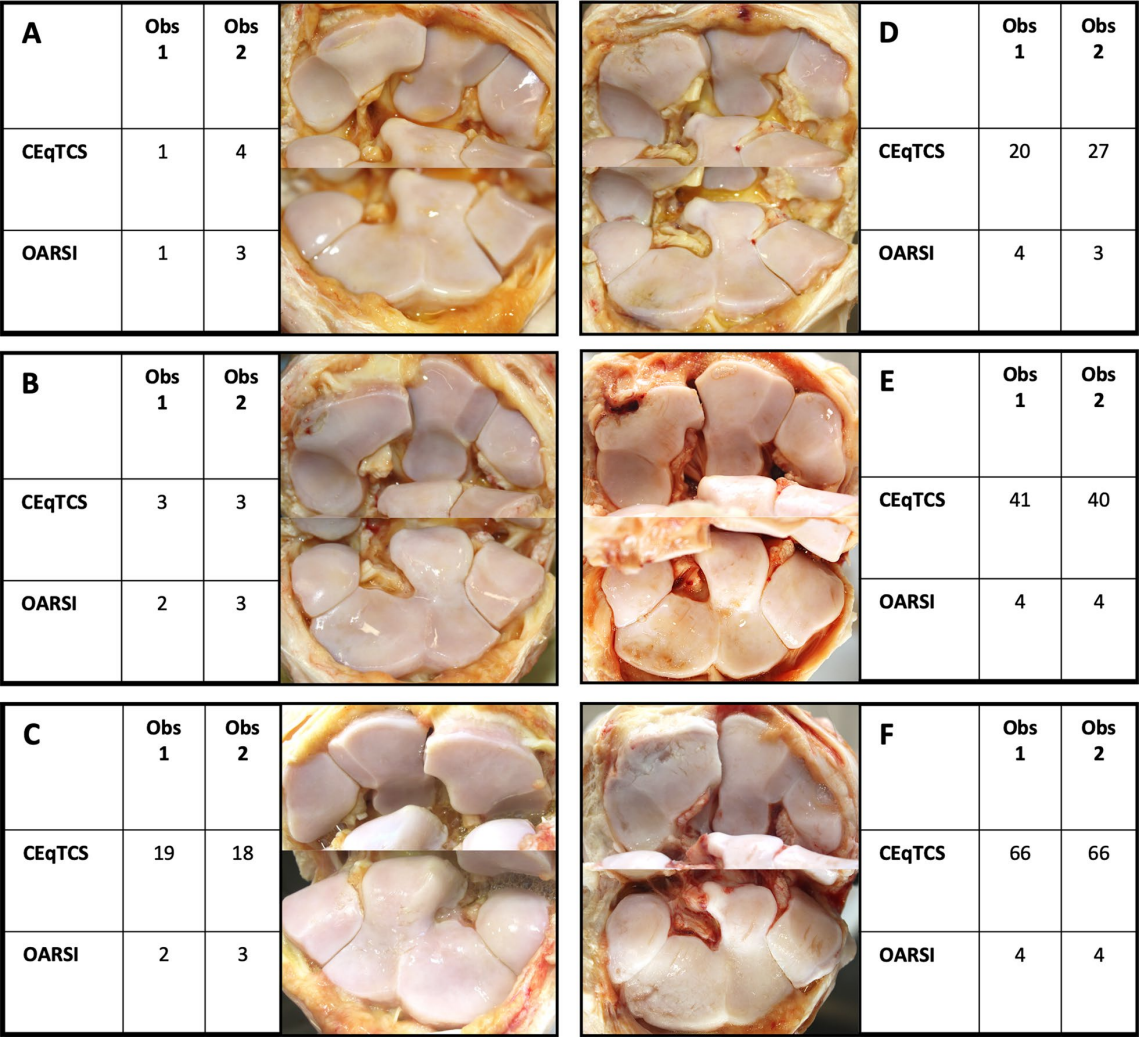
Panel **A**–**F** show the spectrum of pathological changes in the middle carpal joint of six horses. From panel **D**, **E**, and **F** the wide range of the CEqTCS (Copenhagen Equine Total Cartilage Score) within an OARSI (Osteoarthritis Research Society International) grade 4 can be appreciated. Both the OARSI score and the CEqTCS is shown for both observers (Obs 1 and Obs 2)

The new CEqTCS correlated strongly with the OARSI score (p = 7.055e-16, r = 0.94) (Fig. 1C). Although there was good correlation between the two scoring systems, the range of the CEqTCS within each OARSI grade was vast. Observer 1 (OARSI 0: CEqTCS 0–2; OARSI 1: CEqTCS 1–10; OARSI 2: CEqTCS 1–28; OARSI 3: CEqTCS 9–20; OARSI 4: CEqTCS 20–66); Observer 2 (OARSI 0: CEqTCS 0; OARSI 1: CEqTCS 2–5; OARSI 2: CEqTCS 4; OARSI 3: CEqTCS 3–31; OARSI 4: CEqTCS 37–66). Examples are given in Fig. 2.

## Discussion

The new scoring system was easy to use and produced cartilage scoring (CEqTCS) with excellent inter- and intra-observer agreement and consistence. The CEqTCS had excellent correlation with the previously described OARSI system [12], but provided more detailed scoring, especially for the higher scores (Fig. 2). Since any number or size of full-thickness cartilage erosion results in a score of 4, the OARSI system does not allow the observer to distinguish between horses with small or extensive full-thickness cartilage lesions, whereas the new system, which combines scoring of depth, size, and extent/distribution of lesions, allowed us to quantify the pathological changes in more detail (Fig. 2).

The aim of the new scoring system was to create a more sensitive score that would allow us to differentiate OA severity in much more detail than with the OARSI score. This is in line with the suggestions put forward by the inventors of the OARSI score, who stated that “Hopefully, this will serve as a basis for further development and validation of more sophisticated systems” [12]. This level of detail is necessary when the COF model is used for evaluation of potential new drugs for OA treatment [1] or potential biomarkers of OA [13].

For cartilage pathology, each carpal bone was evaluated separately with inspiration from the MRI scoring-system suggested by Smith et al. [6]. The lesion severity and extent was scored separately, in similar pattern of OARSI fetlock score [7]. It is currently not clear whether one/few deep erosions or more widespread superficial erosions have more clinical significance for the patient and encompassing these in one score is therefore of importance. To achieve the CEqTCS of the joint, severity and extent was multiplied with inspiration from the histopathology guidelines provided by Bolam et al. [14], and the sum of the product for every carpal bone was calculated to give the CEqTCS. Evaluating each carpal bone separately gives some advantages. Multiplying extent and severity of cartilage pathology for each bone separately and adding the scores to yield a global cartilage pathology score for the entire joint give researchers the possibility to describe, assess and report disease development and healing more accurately. For instance, it is possible to separately evaluate the 3rd carpal bone, where the kissing lesion opposite the fragment is expected to be located. The cartilage erosions in this site may have a more direct mechanical etiology compared to the more distant pathological changes e.g., in the 2nd or 4th carpal bones or the ulnar carpal bone, which originate from generalized inflammation of the joint. In future refinements of the CEqTCS it may also be possible to subdivide the carpal bones further, e.g., the 3rd carpal bone can be subdivided into the radial and intermediate facets, with the kissing lesion expected to be in the radial facet. However, this was not attempted in this study.

Additionally, assessment of fragment healing separately by a score modified from van den Borne et al. [15] allows for further differentiation of disease severity depending on the goal of the specific study**.** OA affects the entire joint as an organ [16], and evaluation of the articular cartilage alone is not sufficient. Therefore, both the OARSI system and our new system also evaluate the synovial membrane, albeit with some difficulty (moderate intra- and inter-class agreement). The synovium plays an important role in OA [17] and therefore further development and refinement of the synovium score is necessary in order to develop a comprehensive OA score. The CEqTCS, the fragment score and the synovium score were evaluated separately in this study but may be combined into a total pathology score (the CEqCOAS), depending on the purpose of the study.

## Conclusion

The new scoring system provided a highly detailed pathology scoring of OA in the COF model, which allows researchers to detect subtle differences between groups. The scoring system was easy to use and due to the excellent correlation to the existing OARSI score, results obtained with the new scoring system can still be compared to previous studies to some degree.

## Limitations

This scoring system was designed and validated only for experimental, surgically induced OA of the equine middle carpal joint through the COF-model. Further studies are needed to validate this new OA scoring system in naturally occurring OA and in joints other than the middle carpal joint.

## Data Availability

The datasets used and/or analyzed during the current study are available from the corresponding author on reasonable request.

## Abbreviations

ac: Arthrocentesis
COF: Carpal osteochondral fragment (model)
CEqCOAS: Copenhagen Equine Carpal Osteoarthritis Score
CEqTCS: Copenhagen Equine Total Cartilage Score
ICB: Intermediate carpal bone
MCJ: Middle carpal joint
OA: Osteoarthritis
OARSI: Osteoarthritis Research Society International
RCB: Radial carpal bone
UCB: Ulnar carpal bone
2CB: Second carpal bone
3CB: Third carpal bone
4CB: Fourth carpal bone

## References

[1] McIlwraith CW, Frisbie DD, Kawcak CE (2012). The horse as a model of naturally occurring osteoarthritis. Bone Joint Res.

[2] Frisbie DD, Cross M, McIlwraith CW (2006). The quest for a better model: a comparative study of articular cartilage thickness on the femoral trochlea and femoral condyle of species used in pre-clinical studies compared to cartilage thickness in the human knee. Vet Comp Orthop Traumatol.

[3] Foland JW, McIlwraith CW, Trotter GW, Powers BE, Lamar CH (1994). Effect of betamethasone and exercise on equine carpal joints with osteochondral fragments. Vet Surg.

[4] Frisbie DD, Kisiday JD, Kawcak CE, Werpy NM, McIlwraith CW (2009). Evaluation of adipose-derived stromal vascular fraction or bone marrow-derived mesenchymal stem cells for treatment of osteoarthritis. J Orthop Res.

[5] Frisbie DD (1997). Effects of triamcinolone acetonide on an in vivo equine osteochondral fragment exercise model. Equine Vet J.

[6] Frisbie DD, Kawcak CE, Werpy NM, Park RD, McIlwraith CW (2007). Clinical, biochemical, and histologic effects of intra-articular administration of autologous conditioned serum in horses with experimentally induced osteoarthritis. Am J Vet Res.

[7] Frisbie DD, Al-Sobayil F, Billinghurst RC, Kawcak CE, McIlwraith CW (2008). Changes in synovial fluid and serum biomarkers with exercise and early osteoarthritis in horses. Osteoarthr Cartil.

[8] McIlwraith CW, Frisbie DD, Kawcak CE (2012). Evaluation of intramuscularly administered sodium pentosan polysulfate for treatment of experimentally induced osteoarthritis in horses. Am J Vet Res.

[9] Frisbie DD, Ghivizzani SC, Robbins PD, Evans CH, McIlwraith CW (2002). Treatment of experimental equine osteoarthritis by in vivo delivery of the equine interleukin-1 receptor antagonist gene. Gene Ther.

[10] Kawcak CE, Frisbie DD, Werpy NM, Park RD, McIlwraith CW (2008). Effects of exercise vs experimental osteoarthritis on imaging outcomes. Osteoarthr Cartil.

[11] Smith AD (2016). Magnetic resonance imaging scoring of an experimental model of post-traumatic Osteoarthritis In the equine carpus. Vet Radiol Ultrasound.

[12] McIlwraith CW (2010). The OARSI histopathology initiative - recommendations for histological assessments of osteoarthritis in the horse. Osteoarthr Cartil.

[13] McIlwraith CW (2018). Biomarkers for equine joint injury and osteoarthritis. J Orthop Res.

[14] Bolam CJ, Hurtig MB, Cruz A, McEwen BJE (2006). Characterization of experimentally induced post-traumatic osteoarthritis in the medial femorotibial joint of horses. Am J Vet Res.

[15] van den Borne MPJ (2007). International Cartilage Repair Society (ICRS) and Oswestry macroscopic cartilage evaluation scores validated for use in autologous chondrocyte implantation (ACI) and microfracture. Osteoarthr Cartil.

[16] Loeser RF, Goldring SR, Scanzello CR, Goldring MB (2012). Osteoarthritis: a disease of the joint as an organ. Arthritis Rheum.

[17] Sellam J, Berenbaum F (2010). The role of synovitis in pathophysiology and clinical symptoms of osteoarthritis. Nat Rev Rheumatol.

